# Analysis of Immune Responses in Mice Orally Immunized with Recombinant pMG36e-SP-TSOL18/*Lactococcus lactis* and pMG36e-TSOL18/*Lactococcus lactis* Vaccines of *Taenia solium*

**DOI:** 10.1155/2018/9262631

**Published:** 2018-11-18

**Authors:** Bi Ying Zhou, Jun Chao Sun, Xiang Li, Yue Zhang, Bo Luo, Nan Jiang, Mei Chen Liu

**Affiliations:** Department of Parasitology, School of Basic Medical Sciences, Zunyi Medical University, Zunyi, China

## Abstract

Cysticercosis is a cosmopolitan zoonotic parasitic disease infected by larval of *Taenia solium* (*T. solium*). Several drugs for the treatment of cysticercosis, such as praziquantel, albendazole, and mebendazole, have certain toxicity and side effects. Considering that there is no vaccine available, we studied a new vaccine for cysticercosis in this study. The complete *TSOL18* gene and the optimized *SP-TSOL18* gene fragments were obtained using PCR-based accurate synthesis method. The secretory and intracellular recombinant pMG36e-SP-TSOL18/*Lactococcus lactis* (*L. lactis*) and pMG36e-TSOL18/*L. lactis* vaccines of *T. solium* were prepared. Immune responses in mice orally immunized with these two recombinant *L. lactis* vaccines were analyzed by the determination of specific antibodies (IgG, IgG1, IgG2a, and sIgA) in serum, spleen lymphocyte proliferation, and cytokines (IL-2, IFN-*γ*, IL-4, and IL-10) in spleen lymphocyte culture supernatant. Our results showed that, after the first immunization, in these two recombinant *L. lactis* vaccine groups, the levels of serum specific IgG, IgG2a, and IgG1 increased on 14–56 d and reached the highest level on days 42, 42, and 28, respectively. The level of specific sIgA of intestinal mucosa also increased on 14–56 d and reached the highest level on day 42. The level of spleen lymphocyte proliferation increased on 14–56 d and reached the highest level on day 42. The levels of IL-2, IFN-*γ*, IL-4, and IL-10 in spleen lymphocyte culture supernatant increased on 14–56 d and reached the highest level on days 42, 42, 28, and 28, respectively. These results indicated that the recombinant pMG36e-SP-TSOL18/*L. lactis* and pMG36e-TSOL18/*L. lactis* vaccines can induce specific cellular, humoral, and mucosal immune responses in mice with oral vaccination. More importantly, the recombinant pMG36e-SP-TSOL18/*L. lactis* vaccine has a better immune effect. In summary, these results demonstrated the possibility of using *L. lactis* strain as a vector to deliver protective antigens of *T. solium*.

## 1. Introduction

Cysticercosis is a zoonotic parasitic disease that seriously harms human health and is distributed in many developing countries or areas in Latin America, Africa, and Asia [1–3]. A large number of sporadic cases with cysticercosis have been reported in the Southeast and Southern of Guizhou province, such as Kaili, Congjiang, Duyun, and Luodian [4–8]. Surgery and chemotherapy treatment of the disease have several problems, including the limited efficacy, serious side effects, and drug resistance. It is very necessary to develop a safe and effective vaccine against cysticercosis, which can be used in China and other cysticercosis endemic countries [9–12].

TSOL18 is a specific antigen of *Taenia solium* (*T. solium*) oncosphere, which has good immunogenicity and immunoprotection. The *TSOL18* gene is considered to be the most promising candidate vaccine gene and has been studied extensively [13, 14]. *Lactococcus lactis* (*L. lactis*) is an important probiotic in intestine of human and animal. It is generally recognized as safe (GRAS) food grade microorganism and naturally present in milk foods, which has functions of regulating microecological balances, inhibiting tumor growth, reducing cholesterol, delaying aging, and improving immunity [15]. With the development of genetic engineering technology, it has been recently used as a new foreign antigen delivery system and applied to the field of food, vaccines, medicines, health products, and domestic animal breeding industries [16–21]. The objective of this study was to prepare the recombinant pMG36e-SP-TSOL18/*L. lactis* and pMG36e-TSOL18/*L. lactis* vaccines and investigate their induced immune responses in mice. Kunming mice were immunized orally with these two recombinant *L. lactis* vaccines, and then antibodies of serum and intestinal mucosa, proliferation and cytokines of spleen lymphocytes were determined at different time points of postvaccination.

## 2. Materials and Methods

### 2.1. Construction and Identification of Recombinant Plasmids pMG36e-TSOL18 and pMG36e-SP-TSOL18

According to the *TSOL18* gene sequence (Accession No. AF017788), using the *L. lactics* as a host system for gene optimization, the *TSOL18* gene was synthesized using a PAS (PCR-based accurate synthesis) method. The signal secretion protein SP_USP45_ was added at its N-terminus to synthesize the *SP-TSOL18* target gene. Restriction enzyme digestion was performed using *Sac*I and *Hind*III for the *TSOL18* gene fragment and plasmid pMG36e to construct recombinant plasmids pMG36e-TSOL18 and pMG36e-SP-TSOL18. And then transferred them into Top10 competent cells, respectively. Positive clones were selected to perform restriction enzyme digestion and sequencing identification.

### 2.2. Activation of *L. lactis* and Preparation of Competent Cells. *L. lactis*

MG1363 bacteria solution was inoculated in 1 mLG/L-SGM17 (M17 medium + 0.5 M sucrose + 2.5% glycine + 0.5% glucose) liquid culture medium and cultured at 30°C for 72 hours. After obvious turbidity appeared, this culture was inoculated into 5 mLG/L-SGM17 liquid culture medium, incubated at 30°C for 24 hours, and 5 mL of this culture was diluted into 50 mLG/L-SGM17 culture medium and cultivated for 24 hours. Then, 50 mL of the culture was diluted in 400 mL of G/L-SGM17 medium and continually cultured for 3 to 5 hours until the optical density (OD_600_) value of the bacteria solution reached 0.2 to 0.3.

The culture was transferred into a 50 mL centrifuge tube and centrifuged at 4000 rpm at 4°C for 20 minutes, and the supernatant was discarded. The pellet was resuspended in 400 mL of 4°C precooled 0.5 M sucrose containing 10% glycerol, thoroughly shaked, centrifuged, and then discarded the supernatant. Then, the pellet was resuspended in 200 mL of 4°C precooled 0.5 M sucrose containing 10% glycerol and 0.05 M ethylenediaminetetraacetic acid (EDTA), placed in ice water for 15 minutes. The cooled culture was centrifuged again at 4000 rpm at 4°C for 20 minutes, and the supernatant was discarded. The pellet was resuspended by adding 100 mL of 4°C precooled 0.5 M sucrose containing 10% glycerol and shaken well. The culture was centrifuged again, and the supernatant was discarded. The pellet was resuspended in 4 mL of 0.5 M sucrose containing 10% glycerol. After shaking, the final culture was separated into 100 tubes (each containing 40 *μ*L) and placed in an −80°C freezer.

### 2.3. Electrotransformation of *L. lactis* MG1363

The previously obtained plasmids pMG36e-TSOL18 and pMG36e-SP-TSOL18 were separately mixed with competent *L. lactis* MG1363. Both were bathed in ice for 10 minutes and treated with an electronic current. The following electrotransformation parameters were used: a voltage of 2000 V, capacitance of 25 *μ*F, and resistance of 200 Ω. After an initial first pulse, 900 *μ*L of low-temperature GMMC recovery medium (M17 medium + 0.5% glucose + 20 mM MgCl_2_ + 2 mM CaCl_2_) was immediately added. The cultures were placed on ice untouched for 10 minutes, then allowed to resuscitate at 30°C for 2–3 hours. The bacteria solution were centrifuged at 4000 rpm and the supernatant was discarded, and the pellet was concentrated in 100 *μ*L GMMC recovery medium. The solution was then spreaded on 10 *μ*g/mL Erythromycin GM17 agar plates, cultured at 30°C for 2–3 days. Plates were kept in a relatively closed environment, observed for colony growth, and small circular white opaque colonies formed in about one week.

Positive single colony was picked and placed into 1 mL of G/L-SGM17+ 5 *μ*g/mL Erythromycin liquid culture medium, which was then incubated at 30°C for 72 hours until the solution appeared cloudy.

### 2.4. Identification of Recombinant pMG36e-SP-TSOL18/*L. lactis* and pMG36e-TSOL18/*L. lactis* Vaccines

The abovementioned cultured bacteria solution was centrifuged at 10000 rpm for 10 minutes, and the supernatant fluid was discarded. The bacteria solution was centrifuged and washed three times with double-distilled water, and the supernatant fluid was discarded each time. The pellets were resuspended in 30 *μ*L of double-distilled water, placed in a boiling water bath for 10 minutes, then placed in an ice bath for 2 minutes, centrifuging again, and the supernatant was retained for extracting genomic DNA. A 579 bp region of the *T. solium* activated oncosphere *TSOL18* gene, based on the sequence reported by Gauci et al. (1998), was amplified using the forward primer 5′-ATGGTTTGTCGTTTTGCTT-3′ and the reverse primer 5′-TTATGAACGACGAACCTTTTTA-3′. After a positive clone was confirmed, it was prepared for use as an expression strain.

### 2.5. Expression and Identification of TSOL18 Protein

Untransformed *L. lactis* MG1363 bacteria were cultured in GM17 liquid medium. Colonies that were identified as positive were separately selected and inoculated in GM17 liquid medium containing Erythromycin. After stationary culturing at 30°C for 72 hours, the culture was centrifuged at 6000 rpm and 4°C for 15 minutes together with the transformed bacterial solution. The precipitate and supernatant were collected separately for later use. Precooled phosphate-buffered saline solution (PBS) was used to resuspend the precipitate. This culture was then placed in an ice bath and ultrasonicated (300 watts) for 20 minutes, alternating 4 s of ultrasonication with 8 s wait intervals. An equal volume of 2x SDS loading buffer (0.1 mol/L Tris-Cl, pH 6.8, 10% dithiothreitol, 4% SDS, 0.2% bromophenol blue, 20% glycerol) was added, and the culture was then placed in a boiling water bath for 4–8 minutes. The total 20 *μ*L samples were prepared after cooling and then were loaded in SDS-PAGE and Western blot gel electrophoresis plates to separately detect the expression of supernatant and intracellular components.

### 2.6. Animals and Immunity

Eighty specific-pathogen-free (SPF) Kunming mice (40 males and 40 females) were purchased from Experimental Animal Center, Daping Hospital, Third Military Medical University, China (number of animal license SCXK(YU)2012-0005). All mice were 6–8 weeks old, each weighed about 20 g. All experimental procedures involving the mice were performed in accordance with the Regulations for the Administration of Affairs Concerning Experimental Animals approved by the State Council of People's Republic of China.

The Kunming mice were randomly divided into four groups with each group containing twenty mice. The mice in group 1 were immunized with recombinant pMG36e-SP-TSOL18/*L. lactis* vaccine. In group 2, the mice were immunized with recombinant pMG36e-TSOL18/*L. lactis* vaccine. In group 3 and 4, the mice were immunized only with *L. lactis* bacteria and PBS as control groups, respectively. The immunization doses were 3 × 10^9^ CFU [22] and given orally three times with two-week intervals.

### 2.7. Antibody Detection

Four mice were taken from each group on days 0, 14, 28, 42, and 56 after the first immunization. Blood was collected from the orbital vein and let stand for 12 hours at 4°C, then centrifuged at 2000 rpm for 10 minutes to separate the serum. At the same time, mice colons were aseptically removed, cut into pieces, and placed in ice saline solution then ground to homogenate and centrifuged at 3500 rpm and 4°C for 10 minutes. The supernatant was collected and frozen at −20°C for future investigation. The serum specific IgG, IgG1, IgG2a, and intestinal mucosa sIgA [23] were evaluated using the enzyme-linked immunosorbent assay (ELISA) method. The 96-well ELISA plate was coated with 10 *μ*g/mL recombinant TSOL18 antigen. The horseradish peroxidase (HRP) conjugated goat anti-mouse IgG (1 : 100000 dilution), IgG1 (1 : 10000 dilution), IgG2a (1 : 10000 dilution), IgA (1 : 10000 dilution). A diaminobenzidine (DAB) chromogenic substrate was used for staining, and absorbance (OD_450_) values were measured by a microplate reader. Each assay was performed in duplicate.

### 2.8. Preparation of Spleen Lymphocytes

Spleens were also aseptically removed from the four mice per group on days 0, 14, 28, 42, and 56 after the first immunization. The spleen lymphocytes were isolated according to the instructions of the mouse spleen lymphocyte separation kit. Spleen lymphocyte suspension was prepared and adjusted to 5 × 10^6^ cells/mL in RPMI 1640 containing 10% fetal bovine serum. After the number of viable cells was above 90%, penicillin (100 U/mL) and streptomycin (100 U/mL) were added.

### 2.9. Spleen Lymphocyte Proliferation Assay

The cell counting kit CCK-8 detection method was used. Spleen lymphocytes (2 × 10^6^ cells/mL) were dispensed in 24-well culture plates. Three wells were set for each specimen and contained 1 mL stock solution, 1 mL stock solution combining recombinant TSOL18 antigen (10 *μ*g/mL), and 1 mL stock solution combining ConA (10 *μ*g/mL). The cells were incubated in a 5% CO_2_ incubator at 37°C for 48 hours. Two hours before the end of the incubation period, 100 *μ*L of CCK-8 solution was added to each well. Then, absorbance values (OD_450_) were measured for each well with a microplate reader. Each assay was performed in duplicate.

### 2.10. Detection of Spleen Lymphocyte Culture Supernatant IL-2, INF-*γ*, IL-4, and IL-10

Spleen lymphocytes (5 × 10^6^ cells/mL) were dispensed in 24-well culture plates using the method as described in Section 2.9. After the 48-hour incubation, the samples were centrifuged in 4000 rpm for 5 minutes. The supernatant was then collected and assessed for IL-2, INF-*γ*, IL-4, and IL-10 cytokines using a commercial ELISA kit according to the manufacturer's manual. Each assay was performed in duplicate.

### 2.11. Statistical Analysis

Measured data were shown as the mean ± standard deviation (SD). ANOVA models were used for multigroup comparisons, and comparison between groups was performed using the least significant difference method (LSD). Values of *p* < 0.05 were considered to represent statistically significant differences.

## 3. Results

### 3.1. Construction of Recombinant Plasmids pMG36e-TSOL18 and pMG36e-SP-TSOL18

**Figure 1:**
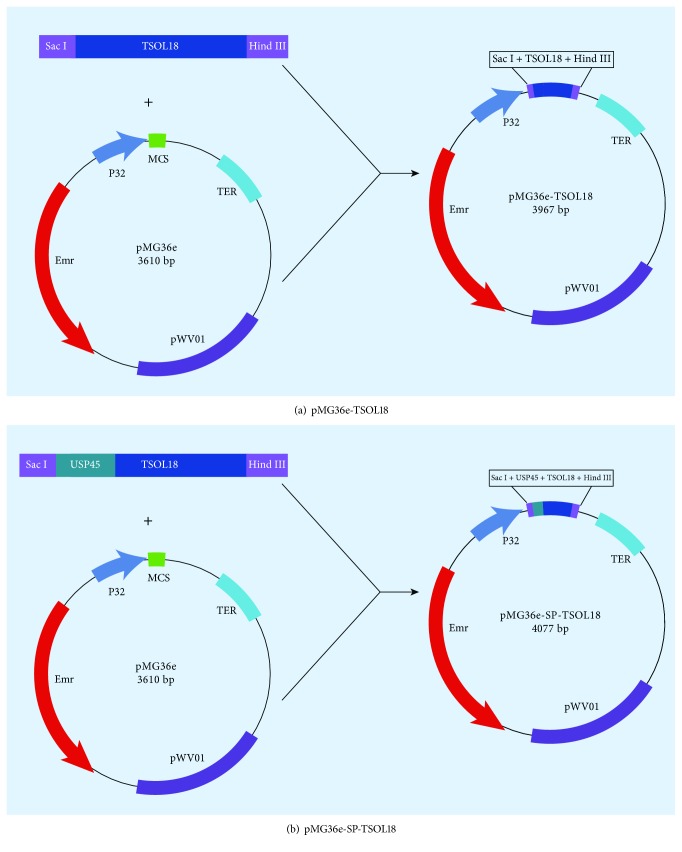
Construction of recombinant plasmids pMG36e-TSOL18 (a) and pMG36e-SP-TSOL18 (b).

Recombinant plasmids pMG36e-TSOL18 and pMG36e-SP-TSOL18 were constructed following Figures 1(a) and 1(b).

### 3.2. Identification of Recombinant Plasmids pMG36e-TSOL18 and pMG36e-SP-TSOL18

**Figure 2:**
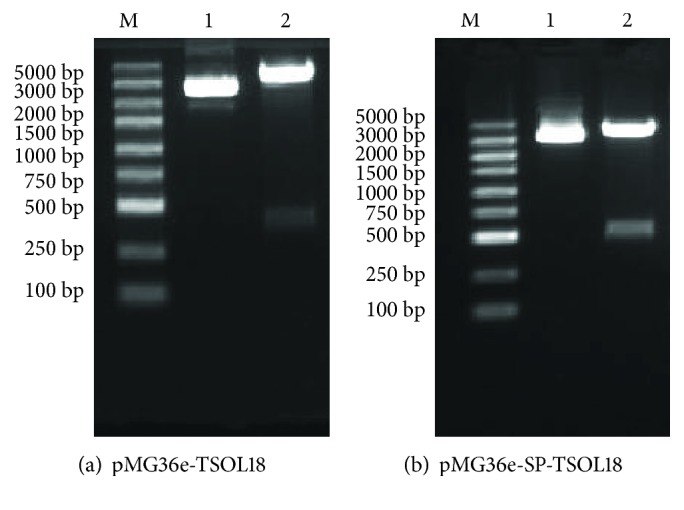
Identification of recombinant plasmid (pMG36e-TSOL18 and pMG36e-SP-TSOL18) by restriction enzyme digestion (*Sac*I and *Hind*III). Lane M, DNA marker; lane 1, plasmid pMG36e; lane 2, production of restriction enzyme of recombinant plasmid pMG36e-TSOL18 (a) and pMG36e-SP-TSOL18 (b).

The amplified *TSOL18* gene fragment and pMG36e vector fragment were digested by restriction enzymes *Sac*I and *Hind*III. The results of 1% agarose gel electrophoresis were shown in Figures 2(a) and 2(b), which were conformed to be the theoretical length. Gene sequencing was performed for recombinant plasmids pMG36e-TSOL18 and pMG36e-SP-TSOL18, which were proved to contain the complete sequences of *TSOL18* gene and pMG36e vector.

### 3.3. Identification of Recombinant pMG36e-TSOL18/*L. lactis* and pMG36e-SP-TSOL18/*L. lactis* Vaccines

**Figure 3:**
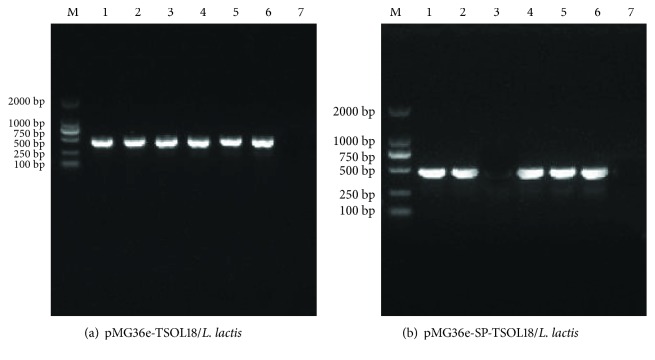
PCR identification of recombinant pMG36e-TSOL18/*L. lactis* and pMG36e-SP-TSOL18/*L. lactis* vaccines. Lane M, DNA marker; Lanes 1–6, PCR products of *L. lactis* MG1363-positive bacteria containing pMG36e-TSOL18 (a) and pMG36e-SP-TSOL18 (b); lane 7, PCR products of *L. lactis* MG1363-negative bacteria.

The culture supernatant of *L. lactis* MG1363 bacteria containing pMG36e-TSOL18 and pMG36e-SP-TSOL18 was used to perform PCR identification. The results showed that lanes 1–6 were the PCR products of *L. lactis* MG1363-positive bacteria containing pMG36e-TSOL18 and pMG36e-SP-TSOL18. Both are consistent with the expected results (see Figures 3(a) and 3(b)).

### 3.4. SDS-PAGE Analysis

**Figure 4:**
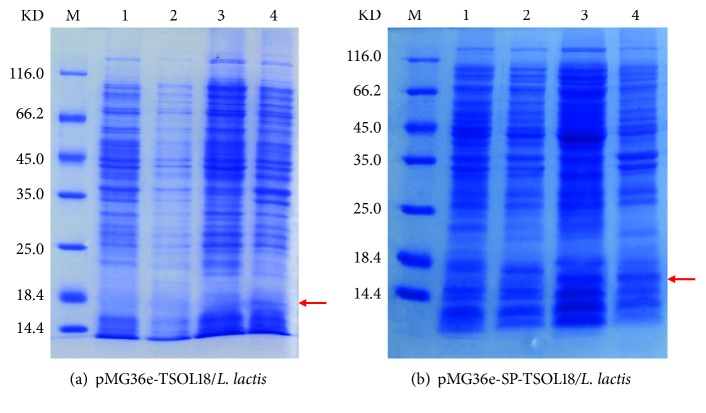
SDS-PAGE analysis of recombinant pMG36e-TSOL18/*L. lactis* and pMG36e-SP-TSOL18/*L. lactis* vaccines. Lane M, protein marker; lane 1, supernatant of MG1363 strain cultured for 72 h; lane 2, precipitation of MG1363 strain cultured for 72 h; lane 3, supernatant of transformation bacteria pMG36e-TSOL18/*L. lactis* (a) and pMG36e-SP-TSOL18*/L. lactis* (b) cultured for 72 h; lane 4, precipitation of transformation bacteria pMG36e-TSOL18/*L. lactis* (a) and pMG36e-SP-TSOL18*/L. lactis* (b) cultured for 72 h.

Positive colonies were selected and inoculated into GM17 liquid medium containing Erythromycin. The colony was cultured for 72 hours at 30°C, then supernatant and precipitation were collected for SDS-PAGE electrophoresis. The results showed that the target protein expression of recombinant pMG36e-TSOL18/*L. lactis* can be observed in around 15 KD of intracellular precipitation. However, no expression has yet been observed in the extracellular supernatant. Recombinant pMG36e-SP-TSOL18/*L. lactis* showed the corresponding target protein expression in both extracellular supernatant and intracellular precipitation (see Figures 4(a) and 4(b)).

### 3.5. Western Blot Identification

**Figure 5:**
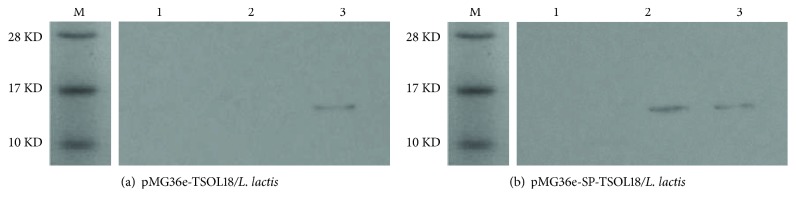
Western blot identification of TSOL18 and SP-TSOL18 protein expression in *L. lactis*. Lane M, protein marker; lane 1, *L. lactis* MG1363-negative bacteria; lane 2, TSOL18 (a) and SP-TSOL18 (b) protein in extracellular supernatant reacted with rabbit-anti-TSOL18; lane 3, TSOL18 (a) and SP-TSOL18 (b) protein in intracellular precipitation reacted with rabbit-anti-TSOL18.

After expression of TSOL18 recombinant protein combined with TSOL18 recombinant rabbit antiserum protein, only the recombinant pMG36e-TSOL18/*L. lactis* appeared around 15 KD reflection band in intracellular precipitation. The recombinant pMG36e-SP-TSOL18/*L. lactis* showed corresponding reaction bands in both extracellular supernatant and intracellular precipitation (see Figures 5(a) and 5(b)).

### 3.6. Serum Specific IgG, IgG1, and IgG2a Levels in Immunized Mice

**Figure 6:**
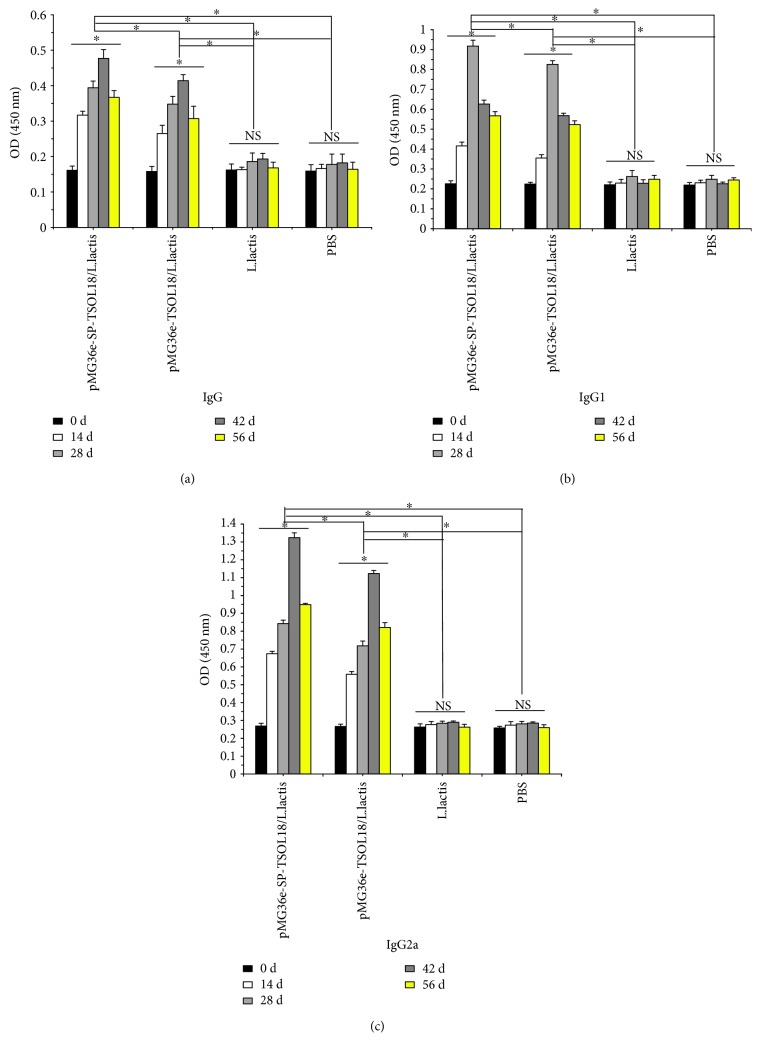
The level of serum specific IgG (a), IgG1 (b), and IgG2a (c) in immunized mice as measured by ELISA, respectively. Serum was obtained on days 0, 14, 28, 42, and 56 after the first immunization. Inset shows the absorbance values of four groups at different time points. ^∗^Represents the difference between groups. *p* < 0.05. NS = nonsignificant.

As compared to the level on day 0, in both recombinant pMG36e-TSOL18/*L. lactis* and pMG36e-SP-TSOL18/*L. lactis* groups, the serum specific IgG, IgG1, and IgG2a levels in mice were increased from 14 to 56 days after the first immunization. Each antibody reached the highest level on days 42, 28, and 42, respectively, which was significantly higher than *L. lactis* and PBS control group (*p* < 0.05). The level of each antibody in recombinant pMG36e-SP-TSOL18/*L. lactis* group was significantly higher than that of recombinant pMG36e-TSOL18/*L. lactis* group (*p* < 0.05) (see Figures 6(a)–6(c)).

### 3.7. Intestinal Mucosa-Specific sIgA Levels in Immunized Mice

**Figure 7:**
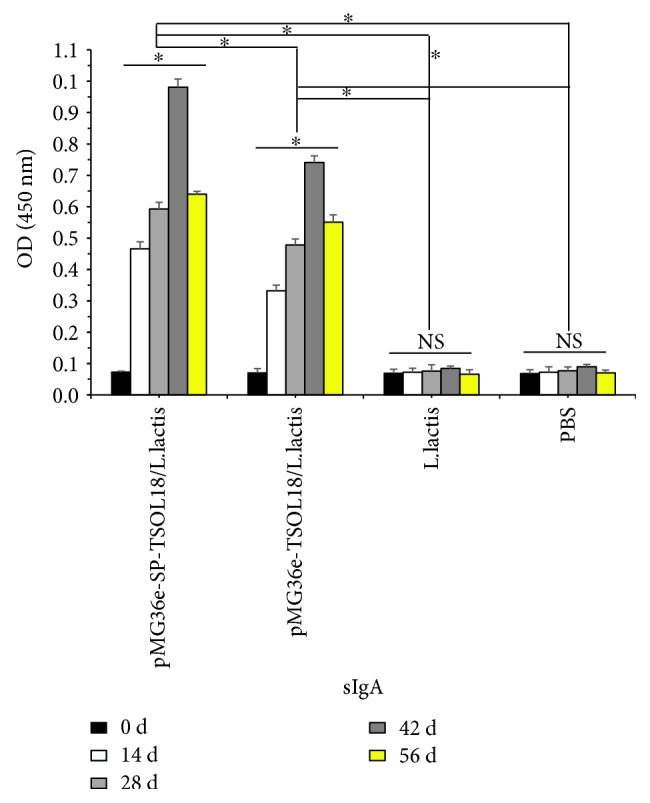
The level of intestinal mucosa-specific sIgA in immunized mice as measured by ELISA. Intestinal mucosa was obtained on days 0, 14, 28, 42, and 56 after the first immunization. Inset shows the absorbance values of four groups at different time points. ^∗^Represents the difference between groups. *p* < 0.05. NS = nonsignificant.

As compared to the level on day 0, in both recombinant pMG36e-TSOL18/*L. lactis* and pMG36e-SP-TSOL18/*L. lactis* groups, the intestinal mucosa-specific secretory IgA (sIgA) levels in mice were increased from 14 to 56 days after the first immunization. sIgA reached the highest level on day 42 which was significantly higher than *L. lactis* and PBS control group (*p* < 0.05). The level of sIgA in recombinant pMG36e-SP-TSOL18/*L. lactis* group was significantly higher than that of recombinant pMG36e-TSOL18/*L. lactis* group (*p* < 0.05) (see Figure 7).

### 3.8. Spleen Lymphocyte Proliferation Levels in Immunized Mice

**Figure 8:**
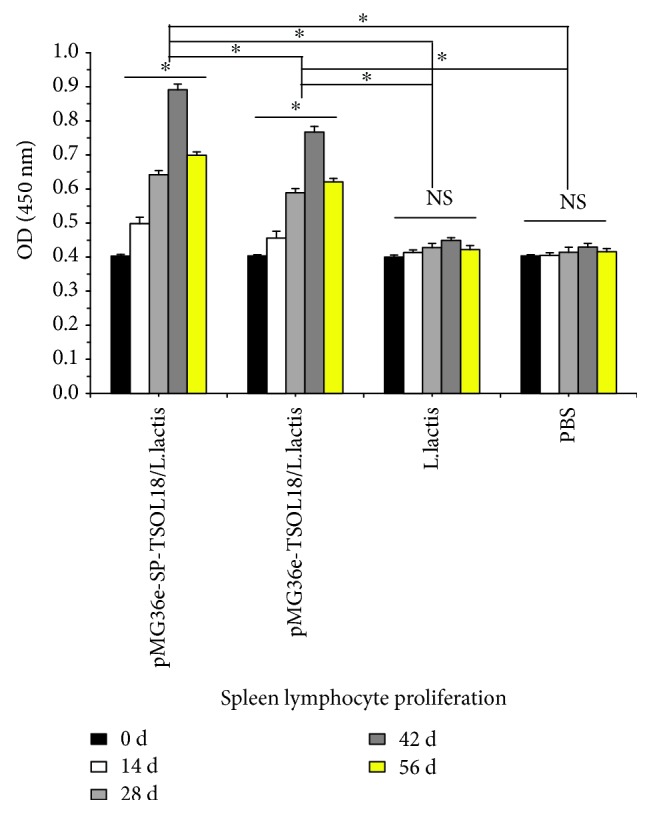
The level of spleen lymphocyte proliferation in immunized mice as measured by CCK-8. Spleen lymphocytes were obtained on days 0, 14, 28, 42, and 56 after the first immunization. Inset shows the absorbance values of four groups at different time points. ^∗^Represents the difference between groups. *p* < 0.05. NS = nonsignificant.

As compared to the level on day 0, in both recombinant pMG36e-TSOL18/*L. lactis* and pMG36e-SP-TSOL18/*L. lactis* groups, the spleen lymphocyte proliferation levels in mice were increased from 14 to 56 days after the first immunization. Spleen lymphocyte proliferation reached the highest level on day 42, which was significantly higher than *L. lactis* and PBS control group (*p* < 0.05). The level of spleen lymphocyte proliferation in recombinant pMG36e-SP-TSOL18/*L. lactis* group was significantly higher than that of recombinant pMG36e-TSOL18/*L. lactis* group (*p* < 0.05) (see Figure 8).

### 3.9. Spleen Lymphocyte Culture Supernatant IFN-*γ*, IL-2, IL-4, and IL-10 Levels in Immunized Mice

**Figure 9:**
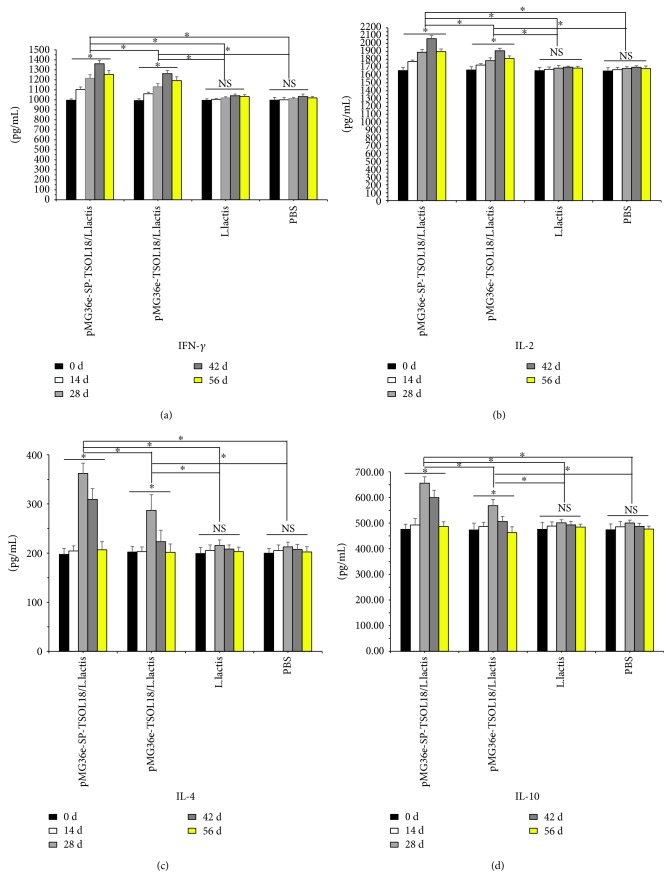
The level of spleen lymphocyte culture supernatant IFN-*γ* (a), IL-2 (b), IL-4 (c), and IL-10 (d) in immunized mice as measured by ELISA. Spleen lymphocytes were obtained on days 0, 14, 28, 42, and 56 after the first immunization. Inset shows the concentration of four groups at different time points. ^∗^Represents the difference between groups. *p* < 0.05. NS = nonsignificant.

As compared to the level on day 0, in both recombinant pMG36e-TSOL18/*L. lactis* and pMG36e-SP-TSOL18/*L. lactis* groups, the levels of IFN-*γ*, IL-2, IL-4, and IL-10 in spleen lymphocyte culture supernatant were increased from 14 to 56 days after the first immunization. Each cytokine reached the highest level on days 42, 42, 28, and 28, respectively, which was significantly higher than *L. lactis* and PBS control group (*p* < 0.05). The level of each cytokine in recombinant pMG36e-SP-TSOL18/*L. lactis* group was significantly higher than that of recombinant pMG36e-TSOL18/*L. lactis* group (*p* < 0.05) (see Figures 9(a)–9(d)).

## 4. Discussion

Cysticercosis is a zoonotic parasitic disease caused by the larvae of *T. solium* in the humans and pigs and led to serious health and economic consequences [24, 25]. There were limitations to medication and surgical treatment [12]. Therefore, it was the best way to eliminate this disease by developing an effective vaccine against *T. solium* infection [26, 27]. Because the eggs of *T. solium* primarily infect hosts through ingestion, *L. lactis* as an oral vaccine for *T. solium* infection may be a more effective as well as practical new vaccine for the prevention and control of cysticercosis [28]. Cysticercosis is caused by *T. solium* eggs or gravid proglottid contamination, and oncospheres are hatched and developed into cysticerci, which would bring great harm to the host. Oncosphere was the key stage in the invasion of host, thus, developing an effective candidate vaccine from oncosphere antigens may be an economic and effective means.

Several recombinant antigens have been expressed and evaluated as potential vaccine candidates such as 45 W, 18 ku, and 16 ku [29–31]. Among these, TSOL18 was the primary vaccine candidate [13, 32], and the *TSOL18* gene was successfully cloned from the *T. solium* oncosphere for the first time. Its coding sequence was highly homologous to other corresponding protective antigens in the tapeworm family, and it was highly conserved among different strains and between different clones [33, 34]. Subsequently, the Chinese researchers Luo et al. successfully cloned the *TSO18* gene of *T. solium*, and the TSO18-GST protein was successfully expressed in *E. coli* [35]. There have been numerous studies on its vaccine potentialities as a recombinant protein, a DNA vaccine, a recombinant yeast vaccine, a recombinant Bacillus Calmette-Guerin vaccine, and a recombinant Bifidobacterium vaccine [36–40], but none of these vaccines has been successfully developed into available ones. *L. lactis* is a good candidate for the delivery of heterologous proteins in foods, which have many advantages such as safety, simplicity, affordability, easiness to prepare, and practicality [41]. As far as the field of parasite is concerned, there has been no report of a recombinant *L. lactis* vaccine of *T. solium*.

The data showed that the signal peptide SPUSP45 derived from *L. lactis* was a major secretory protein and was currently a signal peptide which improved the efficiency of exogenous protein secretion [42, 43]. The secretory expression scheme of this study introduced the fusion of signal peptide SP and the fusion of propeptide fusion LEISSTCDA into the TSOL18 gene to obtain the SP-TSOL18. The intracellular and extracellular (secretory) expressions were designed and expressed in full length, respectively. All of the expected TSOL18 proteins were obtained, and the specific binding to the rabbit antiserum of the recombinant protein of TSOL18 was found. The above results indicate that recombinant pMG36e-SP-TSOL18/*L. lactis* and pMG36e-TSOL18/*L. lactis* were successfully prepared and the TSOL18 protein expressed in extracellular supernatant and intracellular precipitation has specific antigenicity. It is proved that the signal peptide SP and propeptide fusion sequence LEISSTCDA can effectively realize the extracellular expression of TSOL18 and increase the success rate of protein secretion [44, 45]. This lays the experiment foundation for further research on immune responses induced in mice immunized with these two vaccines.

It is important to investigate the immune responses generated in mice by recombinant pMG36e-TSOL18/*L. lactis* and pMG36e-SP-TSOL18/*L. lactis* vaccines. Our results demonstrate that these two recombinant vaccines can induce significant immune responses compared to the levels at the time of nonvaccination at day 0, including antibody isotypes, cytokines associated with activation of both CD4^+^ Th 1 and Th 2 cells, and CD4^+^ T-cell proliferation.

The lymphocyte proliferation test is an important indicator of cellular immunity. As our results showed, spleen lymphocytes showed a strong proliferative response upon stimulation with antigen or mitogen, and the responsiveness in orally immunized mice peaked at day 42, suggesting that recombinant pMG36e-SP-TSOL18/*L. lactis* and pMG36e-TSOL18/*L. lactis* might induce a MHC classII restricted CD4^+^ T cell response. The CD4^+^ T cell may play a role in B-cell differentiation, proliferation, and isotype regulation [46]. Activated CD4^+^ T cells proliferate and differentiate into effector Th cells. This is consistent with the generation of specific cellular immune responses we observed by recombinant *L. lactis* vaccination [47–49]. In addition, lymphocyte proliferation in response to ConA was enhanced substantially. This may be attributed to the fact that ConA produces polyclonal activation of T lymphocytes. Therefore, it is possible that lymphocytes by ConA stimulation showed stronger proliferation response than that for antigen stimulation.

Cytokines and expression of specific isotypes have important role besides regulating the balance between Th1 and Th2 responses [50]. It is known that IL-2, IFN-*γ*, and TNF-*α* are indicators of Th1 response, which promote the production of IgG2a and IgG2b [51, 52], whereas IL-4, IL-5, and IL-10 are indicators of Th2 response, which promote the generation of IgG1, IgG3, and IgE [53–55]. As shown in Figure 9, the stimulation with TSOL18 produced high levels of IFN-*γ*, IL-2, IL-4, and IL-10 in spleen lymphocytes from all immunized groups. As shown in Figure 6, the antibody responses showed a significantly great increase in IgG, IgG1, and IgG2a in orally vaccinated mice than those in nonvaccinated mice. These results demonstrated that cytokines in spleen lymphocytes and antibody isotype in serum from all immunized mice showed that immunization with these two recombinant *L. lactis* vaccines resulted in stimulation of both Th1 and Th2 immune responses. Data showed that the intestinal mucosa is a site that induces an effective mucosal immune response, and the immunization method is simple and easy to operate [56]. sIgA is the main effector molecule of the mucosal immune system, which has a higher content in intestinal mucosa [57]. Our results showed that the specific sIgA level was significantly increased in the intestinal mucosa and these two recombinant *L. lactis* vaccines could induce a mucosal immune response in all immunized mice.

In conclusion, we have demonstrated that an oral live vaccine prepared in this study is capable of inducing specific humoral immune responses, cellular immune responses, and mucosal immune responses in mice and that *L. lactis* is a potential vaccine vehicle to deliver *T. solium* antigens.

## Data Availability

The data used to support the findings of this study are available from the corresponding author upon request.
